# Development of a Slow Loris Computer Vision Detection Model

**DOI:** 10.3390/ani12121553

**Published:** 2022-06-16

**Authors:** Yujie Lei, Ying Xiang, Yuhui Zhu, Yan Guan, Yu Zhang, Xiao Yang, Xiaoli Yao, Tingxuan Li, Meng Xie, Jiong Mu, Qingyong Ni

**Affiliations:** 1 Key Laboratory of Livestock and Poultry Multi-Omics, Ministry of Agriculture and Rural Affairs, College of Animal Science and Technology, Sichuan Agricultural University, Chengdu 611130, China; 201902294@stu.sicau.edu.cn; 2 Farm Animal Genetic Resources Exploration and Innovation Key Laboratory of Sichuan Province, Sichuan Agricultural University, Chengdu 611130, China; 3 College of Information Engineering, Sichuan Agricultural University, Ya’an 625014, China; 202005513@stu.sicau.edu.cn (Y.X.); 201905516@stu.sicau.edu.cn (Y.Z.); 202005509@stu.sicau.edu.cn (Y.G.); 201902158@stu.sicau.edu.cn (Y.Z.); 202005537@stu.sicau.edu.cn (X.Y.); 201902360@stu.sicau.edu.cn (X.Y.); 202105816@stu.sicau.edu.cn (T.L.); 4 Sichuan Key Laboratory of Agricultural Information Engineering, Sichuan Agricultural University, Ya’an 625014, China; 5 College of Life Science, Sichuan Agricultural University, Ya’an 625014, China; xiemeng@sicau.edu.cn

**Keywords:** *Nycticebus*, computer vision, object detection, animal protection, behavior recognition

## Abstract

**Simple Summary:**

Slow lorises are nocturnal primates native to south-east Asia. All the species of slow loris have been listed in Appendix I of the Convention on International Trade in Endangered Species of Wild Fauna and Flora (CITES). It is difficult to artificially detect the slow loris due to its nocturnal habit and venomous bite. This article investigates the feasibility of computer vision for slow loris detection and proposes an improved YOLOv5 algorithm that contributes to formulating an available model for behavior recognition of this endangered taxon.

**Abstract:**

The slow loris (Genus *Nycticebus*) is a group of small, nocturnal and venomous primates with a distinctive locomotion mode. The detection of slow loris plays an important role in the subsequent individual identification and behavioral recognition and thus contributes to formulating targeted conservation strategies, particularly in reintroduction and post-release monitoring. However, fewer studies have been conducted on efficient and accurate detection methods of this endangered taxa. The traditional methods to detect the slow loris involve long-term observation or watching surveillance video repeatedly, which would involve manpower and be time consuming. Because humans cannot maintain a high degree of attention for a long time, they are also prone to making missed detections or false detections. Due to these observational challenges, using computer vision to detect slow loris presence and activity is desirable. This article establishes a novel target detection dataset based on monitoring videos of captive Bengal slow loris (*N. bengalensis*) from the wildlife rescue centers in Xishuangbanna and Pu’er, Yunnan, China. The dataset is used to test two improvement schemes based on the YOLOv5 network: (1) YOLOv5-CBAM + TC, the attention mechanism and deconvolution are introduced; (2) YOLOv5-SD, the small object detection layer is added. The results demonstrate that the YOLOv5-CBAM + TC effectively improves the detection effect. At the cost of increasing the model size by 0.6 MB, the precision rate, the recall rate and the mean average precision (mAP) are increased by 2.9%, 3.7% and 3.5%, respectively. The YOLOv5-CBAM + TC model can be used as an effective method to detect individual slow loris in a captive environment, which helps to realize slow loris face and posture recognition based on computer vision.

## 1. Introduction

Slow loris (*Nycticebus* spp., Lorisidae) [1] is a group of nocturnal primates in Southeast Asia and China. All the species have been listed in Appendix I of the Convention on International Trade in Endangered Species of Wild Fauna and Flora (CITES). The illegal trade and habitat loss have caused a dramatic decline in the wild population of slow lorises [2]. Two species of the slow loris, namely the pygmy (*N. pygmaeus*) and Bengal slow loris (*N. bengalensis*), are distributed in China. Currently, less than 1200 individuals are restricted to 29 forest fragments in Yunnan Province. A large number of individuals were confiscated and kept in captivity due to the illegal harvesting and trade. Animal welfare plays an important role in captive husbandry and animal conservation [3]. Monitoring animal activity is one way to draw conclusions about animal welfare [4]. As the key link of protection and research of slow loris, the observation or identification plays a supporting role in many aspects of slow loris. However, animals often exhibit different behaviors because of people’s presence, which can affect the experimental results. At the same time, manual observation is a labor-intensive task, and it is often impossible for humans to continuously observe all day. Therefore, it is essential to develop an observation method that can work for a long time without human presence.

Object detection is an important research direction in computer vision, where the aim is to accurately identify the class and location of a specific target object in a given image [5]. Compared with the traditional detection methods, computer vision-based object detection has the advantages of speed, versatility and independence of subjective thought.

In recent years, the feature learning and transfer learning capabilities of DCNN (Deep Convolutional Neural Networks) have made significant progress in object detection algorithms, such as feature extraction, image representation, classification and recognition [6,7]. Object detection algorithms based on deep learning are mainly divided into two categories: two stage and one stage. The two stage object detection method first generates regions and then uses the CNNs (Convolutional Neural Networks) for sample classification. The common algorithm is the R-CNN (Region-based Convolutional Neural Network) series. One stage method directly extracts features to predict object class and location. The common algorithm is the YOLO (You Only Look Once) series.

With the large-scale application of deep learning [8] in the field of object detection, the accuracy and speed of object detection techniques have been rapidly improved. Object detection techniques have been widely used in the fields of pedestrian detection [9], face detection [10,11], text detection [12], traffic sign and signal detection and remote sensing image detection. Considerable research has also been conducted in the field of animals. For example, Huang et al. proposed an improved single multi-box detection method for cow condition scoring [13]. Hou et al. developed a CNN-based facial recognition model to identify individual giant pandas [14]. Schütz et al. used YOLOv4 to conduct individual detection and motion monitoring of red foxes [15]. Kalhagen et al. proposed a YOLO-Fish model for fish detection in real-time videos [16].

In order to solve the problems of traditional methods and improve the accuracy of slow loris detection, a novel manually annotated Bengal slow loris detection dataset is established in this study. Based on this dataset, an object detection method is proposed that improves YOLOv5. Improvements are made based on the YOLOv5 network and attention mechanism and deconvolution are introduced in the network. The experiments show that the method proposed in this article has a good effect on the detection of captive slow lorises.

## 2. Materials and Methods

### 2.1. Dataset Acquisition

**Figure 1:**
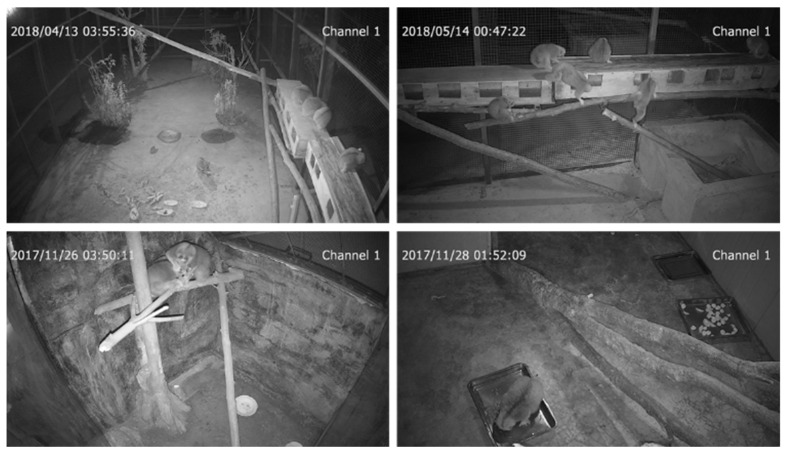
Partial images in the dataset.

The dataset used in this article was obtained from the wildlife rescue centers in Xishuangbanna and Pu’er, Yunnan Province. The information about each rescue center is shown in Table 1. Videos of Bengal slow loris’ activities were continuously acquired by installing night vision monitoring systems (TCNC9401S3E-2MP-I5S and TC-NC9501S3E-2MP-I3S infrared camera, Tiandy Technologies Co., Ltd., Tianjin, China) in their cages. More than 50TB of slow loris activity videos were obtained from April 2017 to June 2018. The obtained videos were extracted with equal frames and one frame was intercepted every three minutes. Because slow lorises are nocturnal animals, most of the videos were taken at night and some images were unclear due to the movement of slow lorises. This part of the data was added to the dataset to enhance the robustness of the dataset. After selection, 1237 images were obtained as the experimental dataset. The dataset is shown in Figure 1. The LabelImg was used to label the dataset. To record the percentage of time the slow loris spent alone and in group activities and thus better determine the degree of harmony of the slow loris group and the degree of an individual slow loris in the group, the status of slow loris was divided into two categories: single and socializing. The dataset was divided into training and validation sets according to 7:3.

### 2.2. Definition of the Slow Loris States

The two states of slow loris, single and socializing, are defined as follows.

Single: The state of activity in which the slow lorises themselves perform behaviors, such as resting and feeding alone.

Socializing: A state in which multiple slow lorises gather to perform behaviors, such as playing, allogrooming or fighting (distance < 0.3 m).

**Figure 2:**
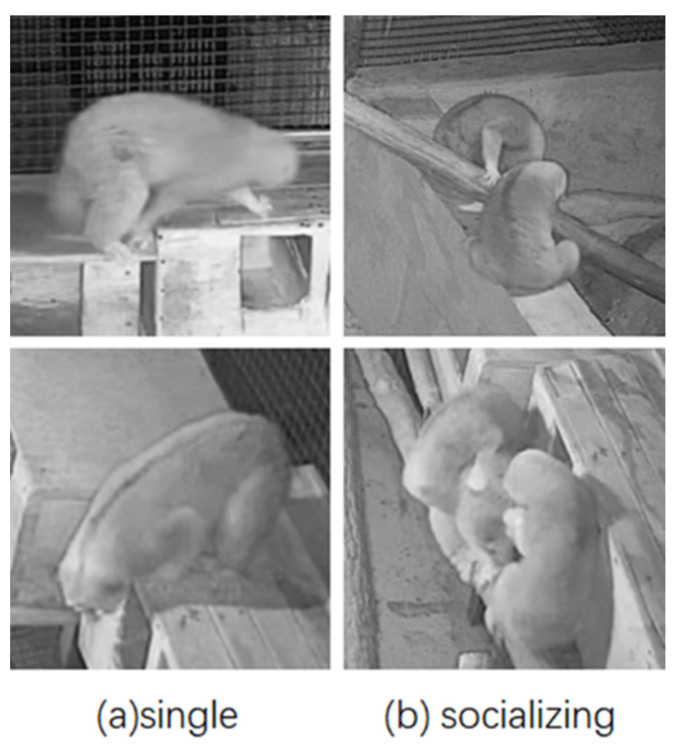
Example diagram of slow loris states.

The two states are shown in Figure 2.

### 2.3. Experimental Environment and Hardware Configuration

The following hardware configurations were used: RTX A4000 16GB graphics card, 12-core Inter (R) Gold 5320 CPU (Central Processing Unit), and 32GB RAM (Random Access Memory). The experimental environments were: Ubuntu (Canonical Ltd., London, UK), CUDA 11.1 (NVIDIA Corporation, Santa Clara, CA, USA), PyTorch 1.8.1 (Facebook Artificial Intelligence Institute, New York, NY, USA), and Python 3.8.0. (Python Software Foundation, New Castle, DE, USA) All experiments in this article were performed on the GPU (Graphics Processing Unit). All experiments were trained using default parameters.

### 2.4. A YOLOv5 Network Introducing Attention Mechanism and Deconvolution

#### 2.4.1. YOLO Series Models

YOLO is another framework, proposed by Redmon et al. in 2016, to resolve the speed problem in the deep learning object detection field after RCNN, fast-RCNN and faster-RCNN, which opened up a new idea of object detection [17,18]. The core idea of YOLO is to replace the two-stage algorithm of RoI (Region of Interest) + target detection with a set of one-stage algorithms of the network and to treat the object detection task as a regression problem; the position of the bounding box and the category it belongs to are directly returned to the output layer.

YOLOv1 uses the method of predefined candidate areas and divides the image into multiple grids. Although each grid can predict multiple bounding boxes, only the bounding box with the highest Intersection over Union (IoU) is selected as the object detection result. Hence, each grid can predict only one object at most. Only one target can be detected if objects occupy a small proportion of the screen; the image contains dense targets and each grid contains multiple targets. Although YOLOv1 is fast in detection, it is not accurate enough in localization and has a low recall rate. Redmon et al. introduced YOLOv2 [19] to improve the localization accuracy and the recall rate. Based on YOLOv1, Redmon et al. made many improvements to improve the mean average precision (mAP) in YOLOv2. However, the problem of identifying a small target is still a difficult task. After that, Redmon et al. proposed YOLOv3, summarizing some of their tentative improvements based on YOLOv2. In YOLOv3 [20], Redmon et al. used the residual model to further deepen the network structure and the Feature Pyramid Networks (FPN) architecture to achieve multi-scale detection. YOLOv3 made progress in the detection of small objects, but the detection of medium and larger sized objects was not ideal. To solve these problems, YOLOv4 and YOLOv5 were proposed later. Based on the original YOLO object detection architecture, many optimization strategies were adopted. There are different degrees of optimization in data processing, backbone network, network training, activation function and loss function. Both YOLOv5 and v4 use Cross Stage Partial Darknet (CSP-Darknet) as the backbone and extract rich informative features from input images [21]. Cross Stage Partial Networks (CSPNet) solve the problem of duplicate gradient information within network optimization. The networks respect the variability of the gradients by integrating feature maps from the beginning and the end of a network stage. Therefore, the number of parameters and floating-point operations per second (FLOPS) values of the model are reduced, improving the speed and accuracy of inference and reducing the model size.

**Figure 3:**
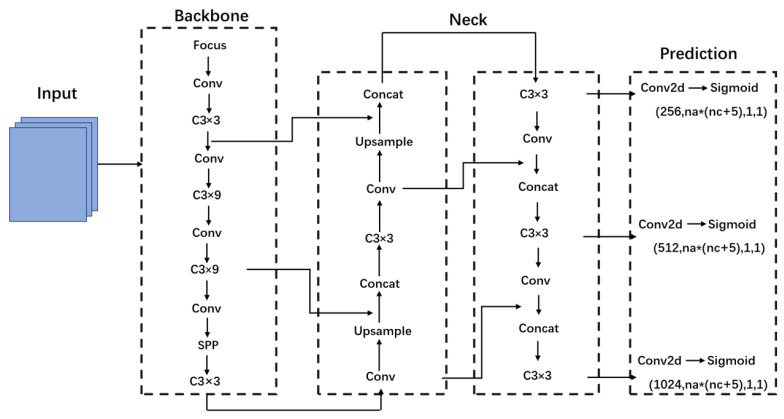
YOLOv5 structure diagram.

The object detection network YOLOv5 has 4 versions, including YOLOv5s, YOLOv5m, YOLOv5l and YOLOv5x. The YOLOv5 model is divided into 4 parts: Input, Backbone, Neck and Output. Figure 3 shows the model structure of YOLOv5. The YOLOv5 is flexible, lightweight and faster with higher accuracy and better small target recognition than the other YOLO series. Therefore, YOLOv5 was selected in this study as the base framework of slow loris detection.

#### 2.4.2. Attention Mechanisms

In recent years, attention mechanism has been widely used in various fields of deep learning [22] and attention models can be seen in various types of tasks such as image processing, speech recognition and natural language processing.

**Figure 4:**
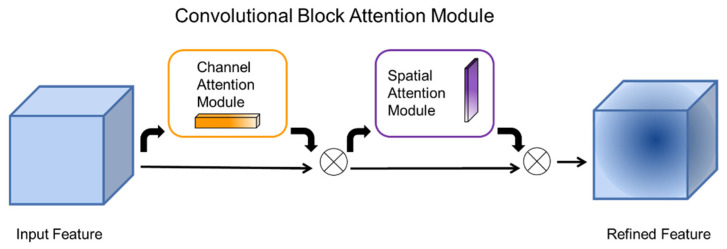
CBAM structure diagram.

The Convolutional Block Attention Module (CBAM) is an attention mechanism module that combines spatial and channel [23]. The CBAM can achieve better results than the attention mechanism that only focuses on channels. The architecture of the CBAM is shown in Figure 4. Because CBAM incorporates spatial attention, it significantly improves the model’s ability to extract key information in space.

In the extraction pre-experiments of this study, the effect of object edge detection did not meet the expectations. After careful analysis, it was found that it was caused by the low clarity in the images and the lack of information about the edges of the objects. Because the CBAM can effectively integrate the spatial information of the images without significantly increasing the convolutional operations, the CBAM is added to the improved YOLOV5 model. The edge blurring problem is effectively solved by improving the information extraction ability of space, which improves the accuracy of the detection model.

#### 2.4.3. Deconvolution

Convolution: Convolution is widely used in the field of image processing [24]. Different convolutional kernels can be used to achieve filtering, edge detection and image sharpening. In a CNN, the features in the image can be extracted by convolutional operation. The low-level convolutional layer can extract some features of the image, such as edges, lines and corners, whereas the high-level convolution layer can learn more complex features from the low-level convolutional layer. In the end, the classification and recognition of the image are obtained.

In the mathematical definition, a convolution is multiplying one variable with another one and adding it to the total sum. In digital image processing, the convolution operation is to let the convolution kernel slide over the image. The gray value of pixels in the image is multiplied by the value on the corresponding convolution kernel, and then all the multiplied values are added as the gray value of pixels in the image.

The following concepts play important roles in convolutional operations:

1. Padding: Before the convolution operation, the boundaries of the original matrix are filled with padding. Specifically, some values are padded on the boundary of the matrix to increase the size of the matrix. 0 is commonly chosen.
2. Stride: When sliding the convolution kernel, start with the top left corner of the input, and step over one column to the left or one row down at a time. The number of rows and columns in each slide is called Stride. During the convolution process, padding is used to avoid information loss and the step size (Stride) is also set to compress part of the information or make the output size smaller than the input one.
3. Channel (number of filters): The number of output channel layers is only related to the number of channels in the current filter.

In addition, there are concepts, such as input graph size, output graph size, and convolution kernel size. The formula of the 2D convolutional output image is calculated as follows (*σ*: the output image size, n×n: the input image size, k×k: the convolutional kernel size, *p*: the size of padding; *s*: the size of the stride):

$$\sigma =\left\lfloor \frac{n+2p-k}{s}\right\rfloor +1$$

After the above, it is easy to see that the size and resolution ratio of the input image will be reduced after a series of convolution operations.

Deconvolution is also known as transposed convolution [25]. The forward propagation process of the convolution layer is the backward propagation process of the deconvolution layer and vice versa. The relationship between the input and output of the deconvolution is defined as:

$$\sigma^{\prime }=s\left(n^{\prime }-1\right)+k-2p$$

It can be seen that deconvolution allows the feature map to go from low to high dimensions [26], which is more random than giving the high-dimensional image directly. The network hyperparameters can be obtained by modifying the transposed convolution kernel. With the successful application of deconvolution on neural network visualization, it has been adopted in various works, such as scene segmentation and generative model.

In the proposed framework, deconvolution is introduced into the YOLOv5 network and is used to restore the feature map obtained by convolution to the pixel space. The patterns the feature map responds to most can be obtained, meaning the features extracted by the convolution operation can be known. The experimental results show that deconvolution effectively improves the accuracy of slow loris detection.

#### 2.4.4. Network Structure

**Figure 5:**
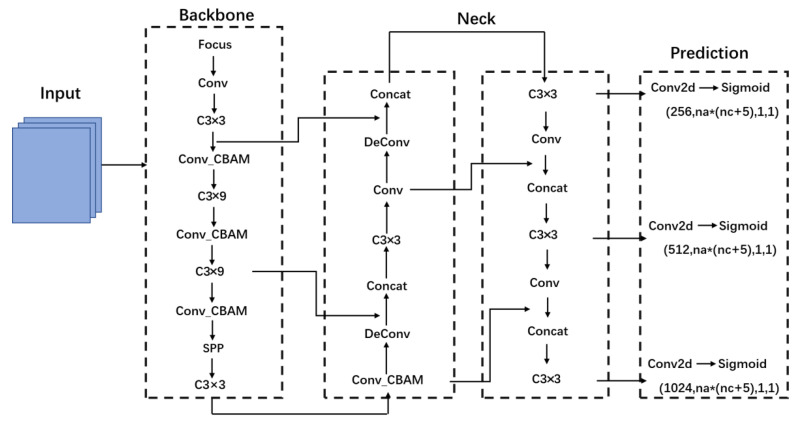
The network structure of YOLOv5-CBAM + TC.

The CBAM and deconvolution are added to the YOLOv5 and YOLOv5-CBAM + TC is obtained with a better detection effect of slow loris. The network structure of YOLOv5-CBAM + TC is shown in Figure 5.

### 2.5. Evaluation Indicators for the Experiment

In this article, precision, recall and mAP are used as evaluation metrics for each model, which are commonly used in the field of object detection. The parameters in the calculation equation of each evaluation metric are defined in Table 2.

Precision is a statistic from the perspective of prediction results. It means the proportion of data is truly positive. That is, the percentage of “right search” or precision is calculated as:

$$\mathrm{precision}=\frac{\mathrm{TP}}{\mathrm{TP}+\mathrm{FP}}$$

Recall is calculated from the real sample set. This means the proportion of positive samples recovered by the model to the total positive samples. That is, the percentage of “complete search”. The Recall is calculated as:

$$\mathrm{recall}=\frac{\mathrm{TP}}{\mathrm{TP}+\mathrm{FN}}$$

The mAP is called mean average precision, which is the average value of AP (average precision) for each category. AP means the area under the precision–recall (PR) curve, and AP is also an indicator of P-R. The better the classifier, the higher the AP value.

The mAP is the average of AP of each category, and this value represents the comprehensive evaluation of the detection target.

mAP@0.5 is the mean average precision when the threshold of IoU is 0.5.

mAP@0.5:0.95 is the average mAP when the threshold of IoU ranges from 0.5 to 0.95 in steps of 0.05.

## 3. Results

### 3.1. Model Comparison

**Figure 6:**
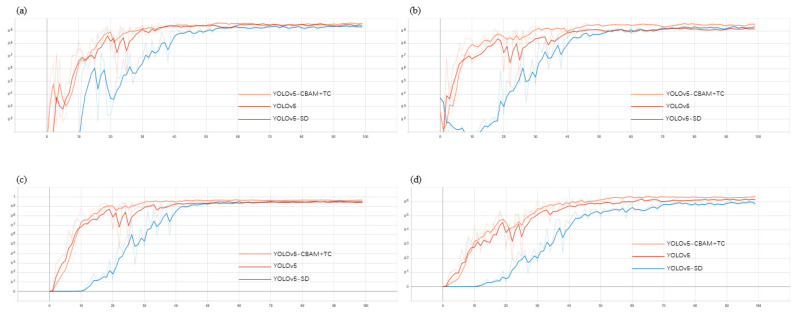
Experimental results of the three models: (**a**) precision; (**b**) recall; (**c**) mAP@0.5; (**d**) mAP@0.5:0.95.

In order to evaluate whether the proposed modified YOLOv5 network can better complete the task of detecting slow loris and whether it is better than the basic YOLOv5, the labeled dataset was used to train 3 networks (YOLOv5, YOLOv5-SD and YOLOv5-CBAM + TC). The dataset contained 1237 images, of which 70% were the training data and 30% were the validation data. The training was performed with 100 epochs, and the experimental results are shown in Figure 6 and Table 3.

### 3.2. Analysis

It can be seen from the experimental results that the YOLOv5-CBAM + TC outperforms the other two models in terms of precision, recall and mAP, and the YOLOv5-SD outperforms the YOLOv5 in terms of recall and mAP, but the precision decreases slightly and is not effective in detecting single slow loris. Therefore, YOLOv5-SD is not considered further and the YOLOv5-CBAM+TC model is selected.

**Figure 7:**
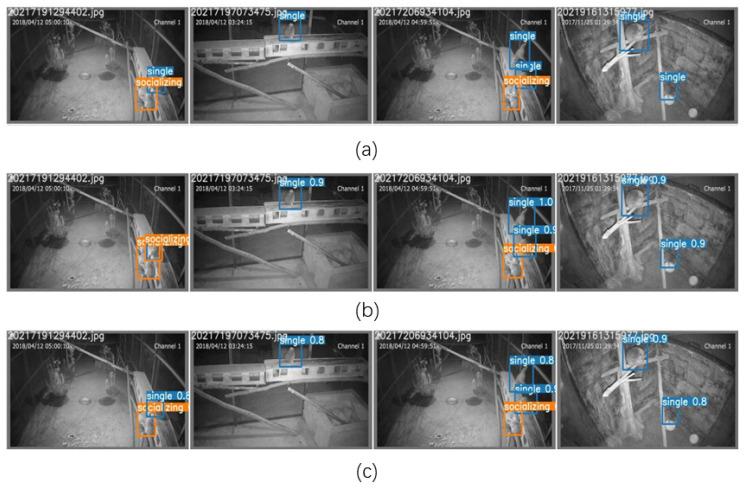
Comparison of YOLOv5-CBAM + TC and YOLOv5 prediction results: (**a**) true labels; (**b**) YOLOv5; (**c**) YOLOv5-CBAM+TC.

Figure 7 shows the true labels of four images randomly selected from the slow loris dataset and the detection effects of the original YOLOv5 and the YOLOv5-CBAM + TC on the four images for lorises in different states.

It can be seen from Figure 7 that there are some slow lorises close to each other but not in a socially active state. The original YOLOv5 model appears to classify them as socially active (the left image in Figure 7b), whereas the improved YOLOv5-CBAM + TC model does not show this phenomenon. The results show that the YOLOv5-CBAM + TC is more suitable for loris detection in the nighttime environment. Embedding CBAM with deconvolution significantly improves the robustness and detection effect of the model, proving the effectiveness of the network.

**Figure 8:**
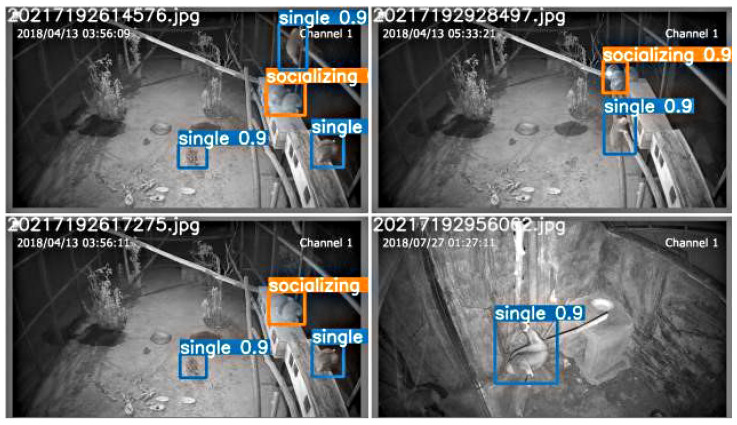
Detection effect of YOLOv5-CBAM + TC.

It can be inferred from Table 4 that the proposed network has a higher mAP than the mainstream detection algorithms. The detection effect of YOLOv5-CBAM + TC is shown in Figure 8.

## 4. Discussion

In this article, the automatic detection model of Bengal slow loris in cages in wildlife rescue centers is studied and the YOLOv5 model is improved. The videos of the nocturnal activity of slow loris are obtained through a night vision monitoring system installed above the cages. The obtained videos are extracted frame by frame and the slow loris detection images are obtained using the improved YOLOv5 model. For the feasibility of the proposed approach, the following are the discussion points:

(1) Because the dataset in this study is collected by a limited number of cameras located on top of the interior of the cage, the observation angle will be limited, and misplacement may occur, leading to incorrect detection results. Therefore, in the subsequent study, the camera positions will be adjusted and the number of cameras will be increased to avoid this problem as much as possible.
(2) In terms of processing speed, YOLOv5 has a high processing speed for the reasons explained in Section 2.4.1. Although the improved YOLOv5-CBAM + TC model has 0.6 MB more than YOLOv5, it only takes 0.1 s to process a single image, which can meet the needs of practical applications.
(3) The datasets used in this study were all human-collected and the collection locations were fixed with relatively simple and single backgrounds. Thus, the accuracy of the model may be reduced in complex environments, such as in the wild. Considering that the scenario currently applied is a case of slow lorises, the scenario in actual application is relatively simple. The authors will continue their research on YOLOv5-CBAM+TS and extend its application scenarios.
(4) In terms of model generalization capability, YOLOv5 adopts a mosaic data enhancement strategy to improve the generalization capability and robustness of the model [27].
(5) Compared with the application of computer vision in the detection of other mammals (such as elephant (Elephantidae) [28] and golden monkey (*Rhinopithecus roxellana*) [29]), the performance of the proposed YOLOv5 CBAM + TC model in the detection of slow loris exceeds the average level and meets the needs of practical applications.
(6) The YOLOv5 CBAM + TC model was operated on a professional server in this study, but it can also be run smoothly on a common laptop, indicating that the model would be economical and practical in a real-world application.

The above discussion demonstrates that the proposed method is exploratory and helpful for slow loris conservation and has an important supporting role in realizing individual identification and behavior recognition of slow loris based entirely on deep learning.

## 5. Conclusions

Although computer vision has been widely applied to animal object detection [30,31], there are still fewer studies on slow loris detection, which significantly affects the protection of endangered species. In this study, computer vision is applied for individual identification and behavioral recognition of slow lorises. After establishing the slow loris dataset, the original YOLOv5 is improved and the YOLOv5-CBAM + TC model is obtained. The proposed model has better precision, recall and mAP than the original YOLOv5, and it has better performance in slow loris detection. The experimental results also demonstrate the effectiveness of the proposed YOLOv5-CBAM + TC. While providing an efficient and accurate method for slow loris detection for scientific researchers engaged in slow loris protection, this study also verifies the feasibility and immense development potential of computer vision in the field of animal protection. The results show that the improved YOLOv5 model has acceptable accuracy and speed in slow loris detection. This study lays a foundation for the welfare improvement of endangered animals, such as slow loris, which have received less attention and are relatively backward in protection measures. In the future, the authors will continue to optimize the YOLOv5-CBAM + TC model, expand its application range from captivity to the wild, and combine it with other deep learning technologies to realize the individual identification [32] and behavior recognition [33] of the slow loris.

## Figures and Tables

**Table 1:** Information about the wildlife rescue centers and the captive Bengal slow lorises.

Captive Site	Xishuangbanna	Puer
Coordinate	22.39276° N, 100.89636° E	22.62198° N, 101.08916° E
Altitude (m)	1060	1600
Annual mean temperature (°C)	17.5	17.5
No. of individuals	9	9
No. of enclosures	1	1
Enclosure size (L × W × H) (m)	5.7 × 4.2 × 3.5	3.5 × 2.1 × 2.0
No. of cameras	3	2

**Table 2:** Parameter definitions.

Confusion Matrix		Predicted Results	
		Positive	Negative
Expected Results	positive	TP ^1^	FN ^2^
	negative	FP ^3^	TN ^4^

^1^ True positive (TP): The prediction result is positive, and the prediction is correct. ^2^ False negative (FN): The prediction result is negative, but the prediction is incorrect. ^3^ False positive (FP): The prediction result is positive, but the prediction is incorrect. ^4^ True negative (TN): The prediction result is negative, and the prediction is correct.

**Table 3:** Experimental information of the three models.

Model	Category	Precision	Recall	mAP@0.5	mAP@0.5:0.95	Model Size
YOLOv5	All	0.936	0.916	0.934	0.609	14.4 MB
	Single	0.955	0.926	0.956	0.554	
	Socializing	0.923	0.906	0.913	0.665	
YOLOv5-SD	All	0.931	0.943	0.95	0.572	16.4 MB
	Single	0.912	0.924	0.955	0.523	
	Socializing	0.951	0.963	0.944	0.622	
YOLOv5-CBAM + TC	All	0.965	0.953	0.969	0.642	15.0 MB
	Single	0.956	0.943	0.964	0.568	
	Socializing	0.974	0.963	0.973	0.716	

**Table 4:** Comparison between mainstream detection algorithms.

Category	YOLOv5-CBAM + TC	SSD	CenterNet	Faster-RCNN
mAP	0.969	0.911	0.889	0.939

## Data Availability

Data sharing not applicable.

## References

[1] Munds R.A., Nekaris K.A.I., Ford S.M. (2013). Taxonomy of the Bornean Slow loris, With New Species *Nycticebus kayan* (Primates, Lorisidae). Am. J. Primatol..

[2] Nekaris K.A.I., Starr C.R. (2015). Conservation and ecology of the neglected slow loris: Priorities and prospects. Endanger. Species Res..

[3] Broom D.M. (1991). Animal welfare: Concepts and measurement. J. Anim. Sci..

[4] Broom D.M. (1988). The scientific assessment of animal welfare. Appl. Anim. Behav. Sci..

[5] Zhao Z.Q., Zheng P., Xu S., Wu X. (2019). Object detection with deep learning: A review. IEEE Trans. Neural Netw. Learn. Syst..

[6] Rawat W., Wang Z. (2017). Deep convolutional neural networks for image classification: A comprehensive review. Neural Comput..

[7] Wang Z., Liu J. (2017). A review of object detection based on convolutional neural network. Proceedings of the 2017 36th Chinese Control Conference (CCC).

[8] LeCun Y., Bengio Y., Hinton G. (2015). Deep learning. Nature.

[9] Dollar P., Wojek C., Schiele B., Perona P. (2011). Pedestrian detection: An evaluation of the state of the art. IEEE Trans. Pattern Anal. Mach. Intell..

[10] Tolba A.S., El-Baz A.H., El-Harby A.A. (2006). Face recognition: A literature review. Int. J. Signal Process..

[11] Hu G., Yang Y., Yi D., Kittler J., Christmas W., Li S.Z., Hospedales T. When face recognition meets with deep learning: An evaluation of convolutional neural networks for face recognition. Proceedings of the IEEE International Conference on Computer Vision Workshops.

[12] Ye Q., Doermann D. (2014). Text detection and recognition in imagery: A survey. IEEE Trans. Pattern Anal. Mach. Intell..

[13] Huang X., Hu Z., Wang X., Yang X., Zhang J., Shi D. (2019). An improved single shot multibox detector method applied in body condition score for dairy cows. Animals.

[14] Hou J., He Y., Yang H., Connor T., Gao J., Wang Y., Zeng Y., Zhang J., Huang J., Zheng B. (2020). Identification of animal individuals using deep learning: A case study of giant panda. Biol. Conserv..

[15] Schütz A.K., Schöler V., Krause E.T., Fischer M., Müller T., Freuling C., Conraths F., Stanke M., Homeier-Bachmann T., Lentz H. (2021). Application of YOLOv4 for Detection and Motion Monitoring of Red Foxes. Animals.

[16] Kalhagen E.S., Olsen Ø.L. (2020). Hierarchical Fish Species Detection in Real-Time Video Using YOLO. Master’s Thesis.

[17] Redmon J., Divvala S., Girshick R., Farhadi A. You only look once: Unified, real-time object detection. Proceedings of the IEEE Conference on Computer Vision and Pattern Recognition.

[18] Girshick R. Fast R-CNN. Proceedings of the IEEE International Conference on Computer Vision.

[19] Redmon J., Farhadi A. YOLO9000: Better, faster, stronger. Proceedings of the IEEE Conference on Computer Vision and Pattern Recognition.

[20] Redmon J., Farhadi A. (2018). Yolov3: An incremental improvement. arXiv.

[21] Bochkovskiy A., Wang C.-Y., Liao H.-Y.M. (2020). Yolov4: Optimal speed and accuracy of object detection. arXiv.

[22] Niu Z., Zhong G., Yu H. (2021). A review on the attention mechanism of deep learning. Neurocomputing.

[23] Woo S., Park J., Lee J.Y., Kweon S. Cbam: Convolutional block attention module. Proceedings of the European Conference on Computer Vision (ECCV).

[24] Gu J., Wang Z., Kuen J., Ma L., Shahroudy A., Shuai B., Liu T., Wang X., Wang G., Cai J. (2018). Recent advances in convolutional neural networks. Pattern Recognit..

[25] Shi W., Caballero J., Theis L., Huszar F., Aitken A., Ledig C., Wang Z. (2016). Is the deconvolution layer the same as a convolutional layer?. arXiv.

[26] Pan J., Sayrol E., Giro-i-Nieto X., McGuiness K., O’Connor N. Shallow and deep convolutional networks for saliency prediction. Proceedings of the IEEE Conference on Computer Vision and Pattern Recognition.

[27] Yao J., Qi J., Zhang J., Shao H., Yang J., Li X. (2021). A real-time detection algorithm for Kiwifruit defects based on YOLOv5. Electronics.

[28] Premarathna K.S.P., Rathnayaka R.M.K.T., Charles J. An Elephant Detection System to Prevent Human-Elephant Conflict and Tracking of Elephant Using Deep Learning. Proceedings of the 5th International Conference on Information Technology Research (ICITR).

[29] Rui S., Xu Z., Ying G., Xinwen Y., Yan C., Yanan H. (2020). Optimized Detection Method for Snub-Nosed Monkeys Based on Faster R-CNN. Laser Optoelectron. Prog..

[30] Verma G.K., Gupta P. (2018). Wild animal detection using deep convolutional neural network. Proceedings of the 2nd International Conference on Computer Vision & Image Processing.

[31] Eikelboom J.A.J., Wind J., van de Ven E., Kenana L.M., Schroder B., de Knegt H.J., van Langevelde F., Prins H.H.T. (2019). Improving the precision and accuracy of animal population estimates with aerial image object detection. Methods Ecol. Evol..

[32] Khan R.H., Kang K.W., Lim S.J., Youn S.D., Kwon O.J., Lee S.H., Kwon K.R. (2020). Animal Face Classification using Dual Deep Convolutional Neural Network. J. Korea Multimed. Soc..

[33] Zuerl M., Stoll P., Brehm I., Raab R., Zanca D., Kabri S., Happold J., Nille H., Prechtel K., Wuensch S. (2022). Automated Video-Based Analysis Framework for Behavior Monitoring of Individual Animals in Zoos Using Deep Learning-A Study on Polar Bears. Animals.

